# Responsiveness of clinical tests for people with neck pain

**DOI:** 10.1186/s12891-017-1918-1

**Published:** 2017-12-28

**Authors:** René Jørgensen, Inge Ris, Carsten Juhl, Deborah Falla, Birgit Juul-Kristensen

**Affiliations:** 1 Department of Physiotherapy, University College South, Degnevej 16, 6705 Esbjerg Ø, Denmark; 2 Department of Sports Science and Clinical Biomechanics, University of Southern Denmark, Campusvej 55, 5230 Odense M, Denmark; 3 Department of Rehabilitation, Copenhagen University Hospital, Herlev and Gentofte, Copenhagen, Denmark; 4 Centre of Precision Rehabilitation for Spinal Pain (CPR Spine), School of Sport, Exercise and Rehabilitation Sciences, College of Life and Environmental Sciences, University of Birmingham, Birmingham, UK

**Keywords:** Neck pain, Clinical testing, Validity, Responsiveness

## Abstract

**Background:**

Responsiveness of a clinical test is highly relevant in order to evaluate the effect of a given intervention. However, the responsiveness of clinical tests for people with neck pain has not been adequately evaluated. The objective of the present study was to examine the responsiveness of four clinical tests which are low cost and easy to perform in a clinical setting, including the craniocervical flexion test, cervical active range of movement, test for the cervical extensors and pressure pain threshold testing.

**Methods:**

This study is a secondary analysis of data collected in a previously published randomised controlled trial. Participants were randomized to either physical training, exercises and pain education combined or pain education only. Participants were tested on the clinical tests at baseline and at 4-month follow-up. An anchor-based approach using Receiver Operator Characteristics (ROC) curves was used to evaluate responsiveness of the clinical tests. The Neck Disability Index was used to discriminate between those who had improved and those who were unchanged at the 4-month follow-up. Minimum Clinically Important Difference (MCID), together with sensitivity, specificity, positive and negative predictive values, in addition to positive and negative likelihood ratios were calculated.

**Results:**

In total, 164 participants completed the 4 month follow up. One-hundred forty four participants were classified as unchanged whereas 20 patients were considered to be improved. Twenty-six participants didn’t complete all of the clinical tests, leaving a total of 138 to be included for analyses. Area Under Curve (AUC) ranged from 0.50-0.62 for the clinical tests, and were all below an acceptable level. MCID was generally large, and the corresponding sensitivity and specificity was low with sensitivity ranging from 20 to 60%, and specificity from 54 to 86%. LR+ (0.8-2.07) and LR- (0.7-1.1) showed low diagnostic value for all variables, with PPV ranging from 12.1 to 26.1 and NPV ranging from 84.7 to 89.2.

**Conclusion:**

Responsiveness of the included clinical tests was generally low when using change in NDI score as the anchor from baseline to the 4-month follow up. Further investigations of responsiveness are warranted, possibly using other anchors, which to a higher degree resemble similar dimensions as the clinical tests.

## Background

Musculoskeletal disorders are the most common form of long-term illness, with neck pain disorders rated as the most frequent complaint in Denmark [1]. It is estimated that 70% of the population will experience neck pain during their lifetime [2], and the one-year prevalence has been reported to be approximately 35% [3].

People with a Whiplash Associated Disorder (WAD), as well as those with idiopathic neck pain often develop neck symptoms lasting for more than 3 months [2, 4]. People with chronic neck pain often present with a variety of other symptoms with a potential large impact on function and quality of life [5]. Several treatment modalities have been evaluated for neck pain, however, there is little evidence to suggest that one treatment is superior to others [6, 7]. The reasons for this may be that 1) the investigated treatment modalities have not been effectively targeted to the right patients, 2) the intervention has not focused on relevant functions, and/or 3) the clinical outcome measures used are not reliable, valid and responsive to detect a change beyond measurement errors.

Clinical tests have been developed for people with neck pain which target the assessment of neuromuscular control and function, such as strength and endurance of the deep neck flexors and extensors [8–11]. Additionally, tests for sensorimotor control such as head repositioning, postural control and head-eye coordination are often included in the clinical examination of patients with chronic neck pain [12–14]. Moreover, since both primary and secondary hyperalgesia is often evident in people with chronic neck pain, tests for pressure pain sensitivity is used increasingly during the clinical examination [15].

In order for a test to be useful in a clinical setting, it needs to be low cost, safe, easy to use and operational within the time frame of a clinical assessment. Furthermore, it has to be reliable, valid and responsive to detect changes. In a previous study, clinical tests including the craniocervical flexion test (CCFT), cervical active range of motion (ROM), gaze stability (GS), smooth pursuit neck torsion test (SPNTT), test for the cervical extensors (CE), balance tests using sway measurements (SWAY) on a Wii balance board and Pressure Pain Threshold (PPT) tests all showed satisfactory reliability, construct and discriminative validity in people with chronic neck pain and in asymptomatic controls [16]. However, the responsiveness, that is the ability of a test to detect a change, remains unknown for these tests. Yet estimating responsiveness of a clinical test is highly relevant in order to evaluate the effect of a given intervention. Three systematic reviews evaluating clinimetrics of cervical muscle function, ROM and cervical sensorimotor control concluded that the responsiveness of such tests was insufficiently described [17–19]. Only PPT tested over the upper trapezius muscle was reported as having acceptable responsiveness when tested in people with neck pain [20]. Therefore, the objective of the present study was to examine the responsiveness of four clinical tests with continuous variables for people with chronic neck pain, which included CCFT, ROM, CE and PPT, since these tests are commonly used in the clinical setting to evaluate the effect of an intervention.

It was hypothesized that the change score of the included clinical tests from baseline to 4-months following an active intervention [21] would correlate with the change in Neck Disability Index (NDI) score over the same time period. It was further hypothesized that all clinical test variables would have an acceptable level of responsiveness.

## Methods

### Study design

The study is a secondary analysis of data collected in a previously reported randomised controlled trial [21]. Participants were recruited between September 2013 and October 2015 from eight different physiotherapy clinics, three hospital units in Jutland and Funen, Denmark, in addition to a municipality on Funen, Denmark. The intervention consisted of 1) physical training, exercises and pain education combined, compared with 2) pain education only, and the protocol has previously been described in detail [22]. The clinical testing procedure followed a standardized protocol [16], and was performed by two experienced musculoskeletal physiotherapists (IR and RJ). Participants were tested at baseline and at the 4-month follow-up. The 4-month follow-up was chosen since it was expected that the intervention of specific exercises would have a measurable effect within this time frame [23]. Prior to the study, examiners were trained to ensure standardization of test procedures.

### Study population

Patients were included according to the following criteria: 18 years or older receiving physiotherapy treatment or having been referred for physiotherapy treatment for chronic neck pain with at least 6 months duration, and reduced physical neck function (NDI-score of at least 10/50) [24], pain primarily in the neck region, and the ability to read and understand Danish.

Patients with neuropathies/ radiculopathies (defined by positive Spurling, cervical traction and plexus brachialis tests) [25], being in an unstable social and/or working situation, pregnancy, known fractures, and depression according to the Beck Depression Inventory (score > 29) [26] were excluded.

Subjects received oral and written information about the project and gave their written informed consent to participate. The Regional Scientific Ethical Committee of Southern Denmark approved the study (S-20100069), and the study conformed to The Declaration of Helsinki 2008.

### Outcome measures

#### Self-reported outcome measures

Demographic data was collected including: age, gender, height and weight and type of accident (if any). Change from baseline to 4 months follow-up on the NDI was used as the criterion measure of clinically important change with higher change scores representing greater recovery. The NDI is a measurement tool covering activities of daily living in people with neck pain. It consists of 10 items with 6 ordinal response categories ranging from 0 to 5, giving a maximum score of 50, with higher scores representing greater disability. The reliability and validity of this measure are acceptable [24, 27, 28]. The reason for selecting NDI as the anchor for responsiveness of the clinical neck tests was to mirror a self-reported variable closely related to neck function.

#### Clinical tests

The tests have been described in detail elsewhere and and have shown acceptable reliability, construct- and discriminative validity [16, 21]. Therefore, they will only be presented briefly.

*Craniocervical Flexion Test (CCFT)* was performed, using a Pressure Biofeedback Unit (Stabilizer; Chattanooga Group, USA), as described by Jull et al. [11]. The subject was asked to perform craniocervical flexion in five incremental stages guided by the pressure sensor. The activation score has six scoring options; 20, 22, 24, 26, 28 and 30 mmHg.

*Cervical Extensor (CE)* test was performed in prone with the head and neck over the edge of the bed. A laser was fixed on top of the subject’s head and was projected to a target. The duration of time the laser beam was kept within the center of the target was measured in seconds (s), as a measure of cervical extensor muscle endurance.

*Range of movement (ROM)* was examined using a bubble inclinometer (Baseline Bubble Inclinometer, Fabrication Enterprises Inc., USA) for flexion/extension and lateral flexion, and with a custom-made equipment for neck rotation. All movements were registered to the nearest degree, except for rotation, which was registered to the nearest 5 degrees.

*Pressure Pain Threshold (PPT)* was examined bilaterally at three sites (cervical spine C5/C6 segment, m. infraspinatus and *M. tibialis* anterior, the latter being a reference value) using a hand-held algometer (Wagner, FPX algometer, USA), and measured in kilogram-force (Kgf). Only data from the cervical spine and *M. tibialis* anterior sites are presented in the current study.

### Statistical analysis

An anchor-based approach using Receiver Operator Characteristics (ROC) curves was performed to examine responsiveness of the clinical tests. ROC-curves were used to evaluate the ability of the clinical tests to detect change in NDI from baseline to the 4-month follow-up. NDI was used to discriminate between those who had improved and those who were unchanged or worsened at the 4-month follow-up. Several studies have reported minimal clinically important difference (MCID) estimates of the NDI for different study populations, ranging from 3.5 to 7.5 for mechanical and non-specific neck pain [29–32]. Based on a systematic review [27], a cutoff greater than 7 change points on the NDI was chosen for the classification of improved, while not-improved were all change scores of 7 or less. Between group differences (improved vs. not-improved) were compared at baseline using independent t-tests for parametric data and Mann-Whitney’s U-test for non-parametric data.

Change scores from the clinical tests were correlated with the change scores of the NDI using Pearsons’s correlation. An acceptable correlation coefficient of 0.30-0.35 was used, as previously recommended for questionnaires when defining the acceptable correlation between anchor and test [33].

Change score from baseline to the 4-month follow-up for each of the clinical tests is the independent variable, and the corresponding ROC curve, plots sensitivity values (true positive) against the 1-specificity values (false positives) with change in NDI of above/below 7 as the dependent variable. Area under the curve (AUC) (95% CI) was used as an indicator of responsiveness, assessing the test’s ability to distinguish patient groups, based on the NDI change scores above/below 7. AUC at 0.50 is considered as no discriminative ability beyond chance, whereas AUC = 1 represents the ability to correctly discriminate all patients. An area 0.7 - 0.8 is considered acceptable, and 0.8 - 0.9 is considered excellent [34].

The MCID was determined as the score, offering the best discrimination between the improved and unchanged/worsened group (greatest sensitivity and specificity). Using the ROC curve the uppermost left-hand corner of the curve represents the optimum condition where both sensitivity and 1-specificity are maximized. Positive and negative predictive values, which represents the chance of finding a true positive change, given a positive test result and a true negative change, given a negative test result [35], in addition to positive and negative likelihood ratios, which is the probability of a positive respective negative test result for a person who has changed divided by the possibility of a positive respective negative test result for a person who hasn’t changed. For all statistical analyses, the STATA statistical package was used (Stata Corp., 2000, Stata Statistical Software: Release 14, College Station, TX, USA).

## Results

A total of 200 patients were included in the original RCT-study [21]. Of these, 164 completed the 4-month follow up and were eligible for the present study. At the 4-month follow-up and according to the described groups classified by their NDI score, a total of 144 (86%) were classified as unchanged, and 20 (14%) as having improved. In the unchanged group, although they completed the 4-month follow up, 26 participants didn’t complete the clinical tests and therefore 118 were included in the analysis.

The two groups did not differ in their baseline demographic characteristics (Table 1), except for duration of symptoms, in which, on average the improved group had symptoms for a significantly longer time compared to those in the unchanged group.

**Table 1:** Baseline demographic characteristics of unchanged and improved groups, presented as mean (sd) and *p*-value

Variable	Total (164)	Unchanged (*n* = 144)	Improved (*n* = 20)	*p*-value
Sex – female n (%)	128 (77)	111 (77)	16 (81)	0.69
Age in years	45.1 (11.7)	45.2 (11.7)	44.9 (12.1)	0.92
Height in cm	170.0 (8.2)	169.8 (8.2)	171.2 (8.6)	0.47
Weight in Kg	77.2 (16.8)	76.8 (16.4)	80.0 (19.5)	0.41
Cause - traumatic onset (%)	97 (59.0)	90 (62.3)	11 (52.7)	0.75
Duration of neck symptoms in months	108.4 (105.8)	99.8 (94.1)	167.2 (155.5)	0.006*
Baseline NDI	21.5 (7.3)	21.3 (7.2)	22.9 (7.8)	0.06

*NDI* Neck Disability Index, *n* numbers; difference was tested using t-test for continuous data and Chi-square for dichotomous

No significant differences in mean change score between unchanged and improved groups were found for any of the clinical tests (Table 2). ROM in neck extension was close to statistical significance (6.34 s (−0.29 to 12.96), *p* = 0.06).

**Table 2:** Change scores, presented with Mean difference (sd), 95% Confidence Intervals and *p*-values for group differences

Variable	Unchanged (*n* = 118)	Improved (*n* = 20)	Mean difference (95% C.I.)	*p*-value
CCFT (mmHg)	1.25 (2.23)	1.80 (2.50)	0.55 (−0.54 to 1.63)	0.32
CE (s)	14.20 (43.59)	24.35 (46.64)	10.16 (−10.90 to 31.21)	0.34
Flexion (°)	−0.11 (12.20)	2.00 (12.70)	3.06 (−2.81 to 8.93)	0.30
Extension (°)	−0.09 (14.02)	6.25 (12.92)	6.34 (−0.29 to 12.96)	0.06
Rotation Right (°)	0.19 (11.51)	4.5 (11.34)	4.31 (−1.18 to 9.79)	0.12
Rotation Left (°)	1.26 (10.76)	3.00 (11.29)	1.74 (−3.44 to 6.92)	0.51
Lateral Flexion Right (°)	−0.36 (9.06)	3.25 (10.10)	3.60 (−0.79 to 8.00)	0.11
Lateral Flexion Left (°)	−0.06 (7.72)	0.40 (5.78)	0.46 (−3.12 to 4.04)	0.80
PPT Tibialis Anterior Right (Kg/f)	−0.36 (1.60)	−0.13 (1.81)	0.33 (−0.55 to 1.01)	0.55
PPT Tibialis Anterior Left (Kg/f)	−0.22 (1.66)	0.08 (1.27)	0.30 (−0.47 to 1.06)	0.45
PPT C5 Right (Kg/f)	0.51 (1.62)	0.16 (2.01)	−0.35 (−2.42 to 1.71)	0.74
PPT C5 Left (Kg/f)	−0.05 (1.50)	0.34 (1.17)	0.39 (−0.31 to 1.09)	0.27

*CCFT* Craniocervical Flexion Test, *CE* Cervical extensors, *PPT* Pressure Pain Threshold, *n* numbers, *CI* Confidence Interval

Correlations between the NDI and the clinical tests were estimated using Pearson’s (r) and ranged from 0.09-0.21, and were below the acceptable level of at least 0.3. Significant correlations were found for ROM in extension and lateral flexion to the right and PPT at C5 left. AUC ranged from 0.50-0.62, (just above discriminate ability beyond chance), and were all below the recommended acceptable level of at least 0.7 (Table 3).

**Table 3:** Correlations (Pearson’s) between change scores of NDI and clinical tests, and AUC with 95% Confidence Interval

Variable	Correlation (r) (95% CI)	AUC (95% C.I.)
CCFT	0.19 (0.03 to 0.35)	0.54 (0.42 to 0.67)
CE	0.09 (−0.08 to 0.25)	0.54 (0.40 to 0.68)
Flexion	0.15 (−0.01 to 0.31)	0.55 (0.41 to 0.69)
Extension	0.21 (0.04 to 0.36)^a^	0.62 (0.50 to 0.75)
Rotation Right	0.09 (−0.08 to 0.25)	0.61 (0.48 to 0.75)
Rotation Left	0.10 (−0.07 to 0.26)	0.56 (0.42 to 0.71)
Lateral Flexion Right	0.20 (0.04 to 0.36)^a^	0.57 (0.44 to 0.70)
Lateral Flexion Left	0.12 (−0.05 to 0.28)	0.52 (0.39 to 0.65)
PPT Tibialis Anterior Right	0.17 (0.01 to 0.33)^a^	0.50 (0.34 to 0.65)
PPT Tibialis Anterior Left	0.14 (−0.30 to 0.30)	0.53 (0.39 to 0.68)
PPT C5 Right	0.14 (−0.03 to 0.31)	0.54 (0.39 to 0.69)
PPT C5 Left	0.21 (0.04 to 0.36)^a^	0.59 (0.44 to 0.74)

*CCFT* Craniocervical Flexion Test, *CE* Cervical extensors, *PPT* Pressure Pain Threshold, *AUC* Area Under Curve, *C.I.* Confidence Interval; ^a^ = Statistical significant

MCID was generally large, and the corresponding sensitivity and specificity were low with sensitivity measures ranging from 20 to 60% (highest for ROM), while specificity ranged from 54 to 86% (highest for CCFT and PPT) (Table 4).

**Table 4:** Minimum Clinically Important Difference, Sensitivity and Specificity, Likelihood ratios and predictive values

Variable	MCID	Sensitivity (%)	Specificity (%)	LR+	LR-	PPV (%)	NPV (%)
CCFT (mm Hg)	2.00	50.0	54.2	1.1	0.9	15.6	86.5
CE (s)	73.0	30.0	85.5	2.1	0.9	26.1	87.7
Flexion (°)	6.0	40.0	74.6	1.6	08	21.1	88
Extension (°)	4.0	55.0	61	1.4	0.7	19.3	88.9
Rotation Right (°)	10.0	40.0	79.5	2.0	0.8	25.0	88.6
Rotation Left (°)	5.0	60.0	55.9	1.4	0.7	18.8	89.2
Lateral Flexion Right (°)	3.0	35.0	63.6	1.0	1.0	14	85.2
Lateral Flexion Left (°)	5.0	20.0	75.6	0.8	1.1	12.1	84.9
PPT Tibialis Anterior Right (Kg/f)	0.83	25.0	84.3	1.6	0.9	21.7	86.6
PPT Tibialis Anterior Left (Kg/f)	0.9	30.0	81.4	1.61	0.86	21.4	87.4
PPT C5 Right (Kg/f)	0.07	45.0	53.5	0.96	1.03	14.5	84.7
PPT C5 Left (Kg/f)	0.48	45.0	78.3	2.07	0.70	25.5	89.1

*CCFT* Craniocervical Flexion Test, *CE* Cervical extensors, *PPT* Pressure Pain Threshold, *MCID* Minimum Clinically Important Difference, *LR+* Positive Likelihood Ratio, *LR-* Negative Likelihood Ratio, *PPV* Positive Predictive Value, *NPV* Negative Predictive Value

Further, LR+ (0.8-2.07) and LR- (0.7-1.1) showed low diagnostic value for all variables [36], and PPV and NPV ranged from 12.1 to 26.1 and from 84.7 to 89.2, respectively.

## Discussion

Responsiveness of the clinical tests evaluated in this study was generally poor when using NDI as an anchor of at least 7 change points for improvement from baseline to the 4-month follow-up in people with chronic neck pain. AUC was low for all variables, likewise all variables (CCFT, ROM, CE and PPT) demonstrated non-satisfactory correlations with NDI, and the MCID was large.

To the best of our knowledge this is the first study to assess responsiveness of clinical tests for people with neck pain since only PPT variables have been evaluated previously in non-chronic neck pain patients [20]. The previous study demonstrated satisfactory responsiveness for PPT measured over the upper trapezius (AUC 0.76; 95% CI: 0.57;0,89) but not for PPT measured over the tibialis anterior (AUC = 0.65; 95% CI:0.46;0.84) [20] which is in contrast to the current findings. There could be several reasons for these contrasting results. Firstly, the study population differs between the two studies, as Walton and colleagues [20] included people with acute or chronic neck pain as opposed to the current study which included only people with chronic pain, and it is likely that differences in severity, symptoms and pain mechanisms affect responsiveness of PPT.

Secondly, although both studies used an anchor based method to measure responsiveness, the anchor differed between studies. Walton et al. used Global Perceived Effectiveness (GPE) in contrast to the current which considered the NDI [20]. Choosing GPE as an anchor for real change as used in some previous studies [29, 31, 37], may be biased due to the subjectiveness of GPE and the questioned reliability and validity of this measure [33, 38]. In the current study with a 4-month follow-up, recall-bias may have been present if GPE was selected, since previous studies have shown GPE to have higher correlation with present than initial status [38, 39]. Moreover, GPE is a generic health related outcome, as opposed to the specific tests evaluated in this study, which is why the NDI, with higher emphasis on self-reported neck function, was selected. However, the correlations between the anchor and the clinical tests were all below the previously set level of acceptance (0.3), indicating that the current clinical tests are not sufficiently covered by changes on the NDI. Since NDI has been critizised for poor sensitivity to longitudinal changes [40] it seems questionable whether large changes on the NDI reflect a longitudinal change in the current study.

The choice of the cut off (at least 7 change points on the NDI) is important for determining the responsiveness. The current cut-off was selected based on the MCID calculated on the NDI in the previously reported systematic review [27]. Choosing another cut-off, for instance a change point of 3 on the NDI, as in some previous studies [31, 32], and/or different cut-offs for the different tests, may have resulted in different estimates. However, post-hoc analysis with a cut-off of 3 change points did not change estimates considerably.

The current MCID variables were all lower than previously reported Minimum Detectable Change (MDC) [16], except for CCFT, meaning that the current calculated MCID could be attributed to measurement error. The current MCID is based on dichotomization of patients as improved and not improved, and does not take into account whether the clinical status on other areas has actually changed. However, PPV and both Likelihood ratios were all below the acceptable levels.

Limitations of this study are the relatively small sample for the improved group (although the total group was large) and the difficulties in identifying the appropriate anchor, that measures the same dimensions as the clinical tests. Using NDI doesn’t seem to be an optimal anchor due to the small group of responders. Since pain is one of the main complaints in this patient group, a measure of pain intensity (eg. Visual Analogue Scale) could be suitable for classifying patients as improved or worsened. The appropriateness of alternative new neck instruments as an anchor remains to be studied in the future. In addition, the clinical tests used may only be classified as semi-objective.

A strength of this present study is that it followed a strict and standardised protocol [21] with a detailed description, and training in the clinical tests and their interpretation. In addition, the study was performed in a clinical setting using simple and low cost clinical tests, previously shown to have satisfactory reliability [16], aiming for high generalizability.

## Conclusion

In conclusion, responsiveness of the included clinical tests (CCFT, ROM, CE and PPT) was generally low when using NDI change score greater than 7 as the anchor point from baseline to a 4-month follow up. A major limitation is the use of NDI as an anchor and further investigations of responsiveness are warranted, possibly using other anchors, for instance pain measures which to a higher degree resemble similar dimensions as the current clinical tests.

## Abbreviations

CCFT: Craniocervical flexion test
CE: Test for cervical extensors
GPE: Global perceived effectiveness
GS: Gaze stability
LR-: Negative likelihood ratio
LR +: Positive likelihood ratio
MCID: Minimum clinically important difference
NDI: Neck disability index
NPV: Negative predictive value
PPT: Pressure pain threshold
PPV: Positive predictive value
ROC: Receiver operator characteristics
ROM: Range of movement
SPNTT: Smooth pursuit neck torsion test
SWAY: Balance tests using sway measurements
WAD: Whiplash associated disorder

## References

[1] Statens Institut for Folkesundhed. Folkesundhedsrapporten, Danmark 2007. 2007; Available at: http://www.si-folkesundhed.dk/upload/forord,_indholdsfortegnelse,forfattere.pdf. Accessed 10 2011.

[2] Cote P, Cassidy JD, Carroll LJ, Kristman V (2004). The annual incidence and course of neck pain in the general population: a population-based cohort study. Pain.

[3] Huisstede BM, Wijnhoven HA, Bierma-Zeinstra SM, Koes BW, Verhaar JA, Picavet S (2008). Prevalence and characteristics of complaints of the arm, neck, and/or shoulder (CANS) in the open population. Clin J Pain.

[4] Carroll LJ, Hogg-Johnson S, van der Velde G, Haldeman S, Holm LW, Carragee EJ (2009). Course and prognostic factors for neck pain in the general population: results of the bone and joint decade 2000-2010 task force on neck pain and its associated disorders. J Manip Physiol Ther.

[5] Ris I, Søgaard K, Gram B, Agerbo K, Boyle E, Juul-Kristensen B (2017). Chronic neck pain patients with traumatic or non-traumatic onset: differences in characteristics. A cross-sectional study. Scand J Pain.

[6] Verhagen AP, Scholten-Peeters GGGM, van Wijngaarden S, de Bie R, Bierma-Zeinstra SMA. Conservative treatments for whiplash. Cochrane Database of Systematic Reviews 2007. Issue 2. Art. No.: CD003338. doi:10.1002/14651858.CD003338.pub3. 10.1002/14651858.CD003338.pub3 PMC8713438 17443525

[7] Hurwitz EL, Carragee EJ, van der Velde G, Carroll LJ, Nordin M, Guzman J (2008). Treatment of neck pain: noninvasive interventions: results of the bone and joint decade 2000-2010 task force on neck pain and its associated disorders. Spine (Phila Pa 1976).

[8] Schomacher J, Farina D, Lindstroem R, Falla D. Chronic trauma-induced neck pain impairs the neural control of the deep semispinalis cervicis muscle. Clin Neurophysiol. 2012;123(7) 10.1016/j.clinph.2011.11.033 22206690

[9] Peolsson A, Kjellman G (2007). Neck muscle endurance in nonspecific patients with neck pain and in patients after anterior cervical decompression and fusion. J Manip Physiol Ther.

[10] Jull G, Kristjansson E, Dall'Alba P (2004). Impairment in the cervical flexors: a comparison of whiplash and insidious onset neck pain patients. Man Ther.

[11] Jull GA, O'Leary SP, Falla DL (2008). Clinical assessment of the deep cervical flexor muscles: the craniocervical flexion test. J Manip Physiol Ther.

[12] Feipel V, Salvia P, Klein H, Rooze M (2006). Head repositioning accuracy in patients with whiplash-associated disorders. Spine (Phila Pa 1976).

[13] Madeleine P, Nielsen M, Arendt-Nielsen L (2011). Characterization of postural control deficit in whiplash patients by means of linear and nonlinear analyses - a pilot study. J Electromyogr Kinesiol.

[14] Treleaven J, Jull G, Grip H (2011). Head eye co-ordination and gaze stability in subjects with persistent whiplash associated disorders. Man Ther.

[15] Chien A, Sterling M (2010). Sensory hypoaesthesia is a feature of chronic whiplash but not chronic idiopathic neck pain. Man Ther.

[16] Jorgensen R, Ris I, Falla D, Juul-Kristensen B. Reliability, construct and discriminative validity of clinical testing in subjects with and without chronic neck pain. BMC Musculoskelet Disord. 2014;15:408 10.1186/1471-2474-15-408 PMC4325947 25477032

[17] de Koning CH, van den Heuvel SP, Staal JB, Smits-Engelsman BC, Hendriks EJ (2008). Clinimetric evaluation of methods to measure muscle functioning in patients with non-specific neck pain: a systematic review. BMC Musculoskelet Disord.

[18] de Koning CH, van den Heuvel SP, Staal JB, Smits-Engelsman BC, Hendriks EJ (2008). Clinimetric evaluation of active range of motion measures in patients with non-specific neck pain: a systematic review. Eur Spine J.

[19] Michiels S, De Hertogh W, Truijen S, November D, Wuyts F, Van de Heyning P (2013). The assessment of cervical sensory motor control: a systematic review focusing on measuring methods and their clinimetric characteristics. Gait Posture.

[20] Walton DM, Levesque L, Payne M, Schick J (2014). Clinical pressure pain threshold testing in neck pain: comparing protocols, responsiveness, and association with psychological variables. Phys Ther.

[21] Ris I, Juul-Kristensen B, Boyle E, Kongsted A, Manniche C, Søgaard K (2016). Does a combination of physical training, specific exercises and pain education improve health-related quality of life in patients with chronic neck pain? A randomised control trial with a 4-month follow up. Man Ther.

[22] Hansen IR, Sogaard K, Christensen R, Thomsen B, Manniche C, Juul-Kristensen B (2011). Neck exercises, physical and cognitive behavioural-graded activity as a treatment for adult whiplash patients with chronic neck pain: design of a randomised controlled trial. BMC Musculoskelet Disord.

[23] Peolsson A, Landen Ludvigsson M, Tigerfors AM, Peterson G (2016). Effects of neck-specific exercises compared to waiting list for individuals with chronic whiplash-associated disorders: a prospective, randomized controlled study. Arch Phys Med Rehabil.

[24] Vernon H, Mior S (1991). The neck disability index: a study of reliability and validity. J Manip Physiol Ther.

[25] Rubinstein SM, Pool JJ, van Tulder MW, Riphagen II, de Vet HC (2007). A systematic review of the diagnostic accuracy of provocative tests of the neck for diagnosing cervical radiculopathy. Eur Spine J.

[26] Carter CL, Dacey CM (1996). Validity of the Beck depression inventory, MMPI, and Rorschach in assessing adolescent depression. J Adolesc.

[27] MacDermid JC, Walton DM, Avery S, Blanchard A, Etruw E, McAlpine C (2009). Measurement properties of the neck disability index: a systematic review. J Orthop Sports Phys Ther.

[28] En MC, Clair DA, Edmondston SJ (2009). Validity of the neck disability index and neck pain and disability scale for measuring disability associated with chronic, non-traumatic neck pain. Man Ther.

[29] Young BA, Walker MJ, Strunce JB, Boyles RE, Whitman JM, Childs JD (2009). Responsiveness of the neck disability index in patients with mechanical neck disorders. Spine J.

[30] Cleland JA, Childs JD, Whitman JM (2008). Psychometric properties of the neck disability index and numeric pain rating scale in patients with mechanical neck pain. Arch Phys Med Rehabil.

[31] Jorritsma W, Dijkstra PU, de Vries GE, Geertzen JH, Reneman MF (2012). Detecting relevant changes and responsiveness of neck pain and disability scale and neck disability index. Eur Spine J.

[32] Pool JJ, Ostelo RW, Hoving JL, Bouter LM, de Vet HC (2007). Minimal clinically important change of the neck disability index and the numerical rating scale for patients with neck pain. Spine (Phila Pa 1976).

[33] Revicki D, Hays RD, Cella D, Sloan J (2008). Recommended methods for determining responsiveness and minimally important differences for patient-reported outcomes. J Clin Epidemiol.

[34] Copay AG, Subach BR, Glassman SD, Polly DW, Schuler TC (2007). Understanding the minimum clinically important difference: a review of concepts and methods. Spine J.

[35] Stratford PW, Riddle DL (2012). When minimal detectable change exceeds a diagnostic test-based threshold change value for an outcome measure: resolving the conflict. Phys Ther.

[36] Jaeschke R, Guyatt GH, Sackett DL (1994). Users’ guides to the medical literature. III. How to use an article about a diagnostic test. B. What are the results and will they help me in caring for my patients? The evidence-based medicine working group. JAMA.

[37] Stewart M, Maher CG, Refshauge KM, Bogduk N, Nicholas M (2007). Responsiveness of pain and disability measures for chronic whiplash. Spine (Phila Pa 1976).

[38] Norman GR, Stratford P, Regehr G (1997). Methodological problems in the retrospective computation of responsiveness to change: the lesson of Cronbach. J Clin Epidemiol.

[39] de Vet HC, Terluin B, Knol DL, Roorda LD, Mokkink LB, Ostelo RW (2010). Three ways to quantify uncertainty in individually applied "minimally important change" values. J Clin Epidemiol.

[40] Lauridsen HH, O'Neill L, Kongsted A, Hartvigsen J. The Danish Neck Disability Index: New Insights into Factor Structure, Generalizability, and Responsiveness. Pain Pract. 2017;17(4):480-93. 10.1111/papr.12477 27440225

